# Advancing Artificial Intelligence for Clinical Knowledge Retrieval: A Case Study Using ChatGPT-4 and Link Retrieval Plug-In to Analyze Diabetic Ketoacidosis Guidelines

**DOI:** 10.7759/cureus.41916

**Published:** 2023-07-15

**Authors:** Ehab Hamed, Anna Sharif, Ahmad Eid, Alanoud Alfehaidi, Medhat Alberry

**Affiliations:** 1 Family Medicine, Qatar University Health Centre, Primary Health Care Corporation, Doha, QAT; 2 Family Medicine, Primary Health Care Corporation, Doha, QAT; 3 Obstetrics and Gynecology, Weill Cornell Medicine - Qatar, Doha, QAT; 4 Fetal and Maternal Medicine, Sidra Medicine, Doha, QAT

**Keywords:** clinical decision tool, clinical decision support system, clinical decision support, chatgpt, large language model, generative ai, artificial intelligence in medicine, chatgpt-4

## Abstract

Introduction

This case study aimed to enhance the traceability and retrieval accuracy of ChatGPT-4 in medical text by employing a step-by-step systematic approach. The focus was on retrieving clinical answers from three international guidelines on diabetic ketoacidosis (DKA).

Methods

A systematic methodology was developed to guide the retrieval process. One question was asked per guideline to ensure accuracy and maintain referencing. ChatGPT-4 was utilized to retrieve answers, and the 'Link Reader' plug-in was integrated to facilitate direct access to webpages containing the guidelines. Subsequently, ChatGPT-4 was employed to compile answers while providing citations to the sources. This process was iterated 30 times per question to ensure consistency. In this report, we present our observations regarding the retrieval accuracy, consistency of responses, and the challenges encountered during the process.

Results

Integrating ChatGPT-4 with the 'Link Reader' plug-in demonstrated notable traceability and retrieval accuracy benefits. The AI model successfully provided relevant and accurate clinical answers based on the analyzed guidelines. Despite occasional challenges with webpage access and minor memory drift, the overall performance of the integrated system was promising. The compilation of the answers was also impressive and held significant promise for further trials.

Conclusion

The findings of this case study contribute to the utilization of AI text-generation models as valuable tools for medical professionals and researchers. The systematic approach employed in this case study and the integration of the 'Link Reader' plug-in offer a framework for automating medical text synthesis, asking one question at a time before compilation from different sources, which has led to improving AI models' traceability and retrieval accuracy. Further advancements and refinement of AI models and integration with other software utilities hold promise for enhancing the utility and applicability of AI-generated recommendations in medicine and scientific academia. These advancements have the potential to drive significant improvements in everyday medical practice.

## Introduction

Remarkable advancements in artificial intelligence and language processing capabilities have propelled AI text generative models, such as ChatGPT, to new heights of performance [1-4]. These models have demonstrated exceptional abilities in providing excellent answers, thanks to their extensive training on diverse corpora during pre-training. However, incorporating these models into scientific academia, particularly medicine, presents challenges and limitations that must be addressed critically [4]. One significant challenge arises from the need for more incorporation of proper citations within the generated text [4]. Linking information to specific sources is crucial for establishing credibility and upholding the integrity of academic writing. Additionally, these models can occasionally generate non-factual information, referred to as "hallucinations" [5], which undermine the reliability and trustworthiness of the generated content. Furthermore, there may be instances where the generated answers lack comprehensiveness, leading to omissions and incomplete outcomes [5]. Addressing these limitations is of utmost importance, necessitating further advancements and improvements in AI text generative models to ensure their suitability for rigorous medical research within scientific academia. Another crucial hurdle lies in identifying dependable sources of medical knowledge that are easily accessible. A recent medical chatbot model incorporated information retrieval from Wikipedia and data from curated medical databases to improve performance [6]. We find national clinical guidelines at the apex of the medical knowledge hierarchy. These guidelines represent the pinnacle of up-to-date, reliable, and practical information that healthcare professionals can readily apply in everyday practice [4]. Several key factors, including the quality and specificity of task instructions, the size and complexity of the task, and the amount and quality of training data, influence the performance of instruction-driven systems in natural language processing (NLP). Previous research has highlighted that providing clear and detailed instructions and breaking down larger tasks into manageable subproblems can enhance the output of large language models [7-9]. In light of these considerations, our study has two primary objectives. Firstly, we evaluate a systematic approach that harnesses the advanced capabilities of ChatGPT-4 in conjunction with external tools. Specifically, we integrate the "URL Reader" plugin to ensure information retrieval from reliable sources, improving reliability and retrieval accuracy. Secondly, in the compilation stage, we prioritize using specific and clear instructions to ensure accuracy and enable proper citations. In conclusion, our study focuses on enhancing traceability, retrieval accuracy, and citation practices by leveraging ChatGPT-4 with the "URL Reader" plugin. We emphasize the importance of using specific and clear instructions and employing successive prompting to break down the task effectively.

## Materials and methods

To ensure a systematic and rigorous approach, this study employed a carefully designed methodology to retrieve, analyze, and compile information exclusively from three internationally recognized guidelines on treating diabetic ketoacidosis (DKA). The selected guidelines were from the United Kingdom [10], Canada [11], and Australia [12], chosen based on their currency and the validity of recommendations. By limiting our source of information to these internationally validated guidelines, we ensured a high-quality and reliable foundation for our study. Our two-step approach employed two prompts to gather and compile information for this study. Firstly, we conducted a data retrieval process focusing on five essential questions about managing DKA: diagnostic criteria, risk factors, signs and symptoms, investigations, and treatment. We used the "Link Reader" plugin to retrieve relevant content, directing each question to a specific guideline and retrieving information through provided URLs. ChatGPT-4 retrieved single-question answers from the designated sources during the first prompt, prioritizing accuracy, truthfulness, and proper citation inclusion. Once three responses were collected for each question, we proceeded to the second step, examining the accuracy of answers against the specified guideline. With one question per guideline at a time, this step-by-step approach ensured a clear source of information and facilitated the examination of answer accuracy. In the second phase, ChatGPT-4 synthesized the information from the three guidelines, eliminating duplicates. The prompt focused on producing comprehensive answers, ensuring proper citations, and providing recommendations that align with the compilation's meaning (Table 1).

**Table 1:** Prompts used to retrieve, compile, and produce recommendations.

Prompt 1	Prompt 2
As a scholar experienced in research on [Topic], your expertise is required to provide accurate and comprehensive answers for complete understanding. If a cohesive text is available, copy it word for word. Question: [Question]. Persona: The Scholar. Research Paper URL: [Link]. Instructions to Attain Unparalleled Accuracy in Information Retrieval: Provide a thorough answer that incorporates all the information available in the research paper. Search different sections of the paper to address each question, using your comprehensive understanding of the research. Ensure precise and complete responses to all questions by applying rigorous analysis and scrutiny. Address all relevant criteria mentioned in the research paper, supporting your answers with evidence and references from the text. If the answer is not present or is inconclusive based on the available information, acknowledge this and state that the answer is not available. Make sure your response is accurate and verifiable, with every word accurately representing the information in the paper. Answer: [Leave this section blank]."	As a medical academic experienced in the field, your expertise is required to provide accurate and comprehensive recommendations for the following question based on the answers above. You will only answer from the three guidelines provided. Question: [Question]. Task: Write a paragraph that compiles the answer, ensuring no duplication. If multiple guidance points are present, select the most common one and cite the sources. Any additional variations should be referenced using numbered in-bracket citations (e.g., [1], [2]). You will then provide a recommendation, ensuring clarity and cohesion. Your recommendations should maintain the meaning of the original content and strive to keep the wording as close to the original as possible, especially when reporting numerical values. Instructions: Comprehensive Answer Compilation: Analyse the question and its corresponding answer from the three guidelines provided. Compile a paragraph combining the information from the answers, avoiding duplication. If multiple guidance points exist for a specific question, choose the most common one and note any additional variations. Provide individualized citations (e.g., [1], [2]) for each variation, referring to the respective guidelines. Recommendation Development: Develop a recommendation at the end of each paragraph or subsection following the compiled answer. Ensure the recommendation aligns with the original content's meaning and keeps the wording as close to the original as possible. Maintain clarity and cohesion, logically connecting the recommendation to the preceding content. Accuracy and Integrity: Preserve accuracy and integrity in your reporting, maintaining the content's original meaning. Be careful when reporting numerical values, ensuring their precise representation without alteration. Note: Focus only on the provided guidelines and their corresponding questions and answers. Avoid introducing personal opinions or analysis. Your goal is to compile and present information accurately and comprehensively, providing meaningful recommendations."

Throughout the study, utmost attention was given to the accuracy and integrity of the information produced and cited. The results are presented as they were produced by the AI model, allowing verification and validation. The focus remained solely on presenting the final recommendations and related questions and answers without personal opinions or subjective analysis. This study provided a potential framework for automating medical text synthesis by implementing this systematic approach and leveraging advanced AI tools. The goal is to benefit medical professionals and patients by offering reliable and consolidated information from multiple reputable sources.

## Results

ChatGPT-4, with the "Link Reader" plug-in, gathered relevant information and generated well-informed recommendations. The clear instructions provided, the systematic approach adopted, and allowing the model sufficient time to create responses all contributed to the positive results achieved in our study. We present the final recommendations in Table 2, and the more comprehensive results of our study can be found in the Appendix section (Appendix 1).

**Table 2:** AI-produced recommendations. AI: Artificial Intelligence.

Question	Recommendation
What are the diagnostic criteria for Diabetic Ketoacidosis (DKA)?	The most common diagnostic criteria for DKA can be summarised as follows: Hyperglycemia (typically blood glucose level >11 mmol/L), metabolic acidosis (typically venous pH <7.3 and/or bicarbonate <15 mmol/L), and the presence of ketones in the blood or urine. It is important to note that these criteria may vary slightly depending on specific patient circumstances, such as the use of SGLT2 inhibitors. It should always be interpreted in the context of the patient's overall clinical picture.
What are the risk factors associated with Diabetic Ketoacidosis (DKA)?	The most common risk factors for DKA can be summarised as follows: a new diagnosis of diabetes mellitus, insulin omission, infection, use of certain medications, and stress conditions such as surgery, trauma, and infections. It is important to note that these risk factors may vary slightly depending on specific patient circumstances, such as the use of SGLT2 inhibitors, and should always be interpreted in the context of the patient's overall clinical picture. It is recommended that healthcare professionals inform all patients commencing SGLT2 inhibitors about the risks of DKA/euglycemic DKA, including potential symptoms and signs, and provide management advice [6].
What are the signs and symptoms of DKA?	The most common signs and symptoms of DKA can be summarised as follows: Polyuria, polydipsia, nausea and vomiting, abdominal pain, shortness of breath, confusion or difficulty paying attention, fatigue or extreme tiredness, hyperglycaemia, acidosis, and the presence of blood or urinary ketones. It is important to note that these symptoms and signs may vary slightly depending on specific patient circumstances and should always be interpreted.
What are the investigations for DKA?	Given DKA’s complexity and potential severity, conducting a comprehensive set of investigations is crucial to ensure accurate diagnosis and effective treatment. These investigations should include, but not be limited to, the diagnostic criteria for DKA, assessment of plasma levels of electrolytes, anion gap, plasma glucose, creatinine, osmolality, beta-OHB, blood gases, serum and urine ketones, fluid balance, and level of consciousness. Additionally, precipitating factors and complications should be diagnosed and treated. Blood ketone testing is preferred, and the equipment should be made available for medical practices and 'at-risk' patient use. The main aim in treating DKA is to progressively normalise the blood pH and clear the body of excessive ketones, achieved by aggressive fluid replacement and insulin therapy. These investigations should be conducted under the supervision of a healthcare professional, and the specific investigations may vary based on the individual's condition and the healthcare provider’s clinical judgment.
What is the treatment for DKA?	Given DKA’s complexity and potential severity, conducting a comprehensive set of treatments is crucial to ensure effective management. These treatments should include fluid resuscitation, insulin administration, avoiding hypokalemia and rapidly falling serum osmolality, and searching for precipitating causes. Blood ketone testing is preferred, and the equipment should be made available for medical practices and 'at-risk' patient use. The main aim in treating DKA is to progressively normalise the blood pH and clear the body of excessive ketones, achieved by aggressive fluid replacement and insulin therapy. These treatments should be conducted under the supervision of a healthcare professional, and the specific treatments may vary based on the individual's condition and the healthcare provider’s clinical judgement.

During the first phase of our study, we observed a consistent level of accuracy in the responses generated by the first prompt. For instance, when addressing the diagnostic criteria for DKA, the answers consistently aligned with the recommendations provided in the guidelines. The retrieved numbers always matched the specified thresholds across different guidelines, whether blood sugar levels were >14 mmol/L or >11 mmol/L. Similarly, when exploring the signs and symptoms of DKA, the generated answers consistently reflected those mentioned in each guideline. The compilation process, initiated with the second prompt, yielded highly accurate results. This ensured the inclusion of relevant information and appropriate citations throughout the compilation. The compiled answers were significantly more extensive than the final recommendations, further reinforcing the robustness of our study's outcomes. Notably, the generated recommendations demonstrated excellent quality, affirming the overall success of our research. During our study, we encountered some challenges accessing specific web pages and ensuring direct alignment with the intended questions. For instance, one of the sources provided diagnostic criteria instead of particular signs and symptoms, while another focused primarily on investigations related to the diagnosis of DKA. These challenges were addressed during the compilation stage, as ChatGPT-4 compiled the answers from the three sources, ensuring a comprehensive representation of the information. Additionally, we observed "memory drift," where the model generated answers that deviated from the original questions, requiring us to restart the process. However, it is essential to note that these limitations were relatively minor and did not significantly impact the overall findings of our study. It is essential to highlight that the results discussed in this study pertain specifically to the ChatGPT-4 model and the associated plug-in. These resources are currently available through paid access, which restricts their usage to a more limited audience, limiting their accessibility to the broader public. In conclusion, despite the difficulties and limitations encountered during our study, we maintained a commendable level of accuracy, reliability, and quality in the generated content and compiled recommendations. The output can be considered as a draft suitable for expert review.

## Discussion

Our study aimed to assess a systematic methodology specifically developed to enhance the traceability and retrieval accuracy of ChatGPT-4, an advanced AI text generative model. By retrieving, analyzing, and synthesizing information solely from three internationally recognized guidelines on the treatment of DKA, our objective was to test the hypothesis that adopting a single-source, single-question approach from reliable sources would significantly improve the accuracy and traceability of the information generated by the model. This research contributes to the growing body of literature on successfully utilizing ChatGPT-4 for medical data retrieval, extraction, and compilation. The findings shed light on the potential of employing a focused methodology, supported by reliable sources and systematic techniques, to enhance the accuracy and traceability of information generated by ChatGPT-4 when applied to high-quality medical knowledge, such as medical guidelines. These results affirmed our expectations and underscored the importance of a meticulous methodology in enhancing AI-generated text's trustworthiness, verifiability, and applicability, particularly within academic and medical research. The remarkable accuracy demonstrated by the first prompt in reporting ensured the inclusion of relevant information accompanied by proper citations. Additionally, the subsequent recommendations and the synthesized data from the second prompt exhibited a commendable level of accuracy, reliability, and quality. These findings underscore the necessity of prioritizing accuracy, truthfulness, proper citation, and alignment with the original text throughout the synthesis process. By focusing on these critical elements, we generated content that closely adhered to the desired standards of reliability and traceability. While our study encountered particular challenges and limitations, such as difficulties accessing specific web pages and occasional alignment issues with the intended questions, our approach remained robust, resulting in comprehensive answers. Instances of 'memory drift' were observed but did not significantly impact our findings. Instead, we recognized these limitations as opportunities for further refinement of the AI model and its application, bolstering the process rather than impeding it. Our study provides compelling evidence for the utility and potential of AI text generative models in medical research, as long as their limitations are continuously addressed and mitigated. Our work represents a significant stride toward a broader goal. As AI continues to develop and advance, it is imperative to conduct further research to refine these tools and amplify their value within academic settings, particularly in medicine. Our methodology offers a promising framework for automating medical text synthesis and showcases an innovative approach to utilizing ChatGPT-4 and the 'Link Reader' plug-in. Given the rapid pace of development, additional alternatives will likely emerge in the coming weeks and months. These tools can serve as dependable chatbots, utilizing international guidelines to generate comprehensive answers rapidly. Moreover, our method can be adapted to access multiple international guidelines simultaneously, broadening the scope and strengthening the reliability of the gathered information. Beyond the specific context of our study, the systematic process of selecting reliable resources, formulating precise questions, and compiling consensus-driven information can be adapted across various domains within medical research and other scientific fields. The versatility of this approach highlights the expansive possibilities offered by advanced AI tools. We anticipate that the direct beneficiaries of our work will be medical professionals and patients, who will gain access to reliable, consolidated, and easily digestible information from numerous reputable sources. Additionally, the indirect benefits of our research extend to potentially revolutionizing how scientific knowledge is collated, presented, and interpreted. The content generated in our study, deemed suitable for expert review, not only represents a notable advancement in AI-assisted medical research but also showcases the broader transformative potential of AI in scientific inquiry. Our study marks a pivotal step toward enhancing AI-generated text's reliability, traceability, and utility within academic discourse. AI has revolutionized clinical decision-making in several areas. Treatment planning benefits from machine learning algorithms that suggest personalized treatment plans based on disease severity [1]. Clinical decision support (CDS) tools powered by AI analyze extensive data to improve diagnoses, suggest subsequent courses of action, and enhance the efficiency of the care team [3]. Remote patient monitoring (RPM) using AI enables real-time monitoring, risk detection, and optimization of healthcare resources [13]. AI algorithms excel in analyzing medical images for accurate and prompt disease diagnosis [14]. AI tools that synthesize data for tailored recommendations facilitate personalized patient care planning. Predictive models integrated into CDS systems can identify at-risk patients and provide evidence-based recommendations. The potential of AI-based CDS tools to enhance patient care and improve healthcare delivery is vast. These tools can facilitate clinical decisions, promote evidence-based care, and improve patient health. However, despite their advances, we believe generative AI will contribute significantly to their advancement, and integrating these tools into clinical settings could be faster [15]. As AI continues to advance and evolve, with ongoing refinement, we firmly believe that AI text generative models like ChatGPT-4 can become indispensable tools for medical professionals and researchers, making significant contributions to advancements in medical research.

## Conclusions

In conclusion, this study aimed to enhance the traceability and retrieval accuracy of ChatGPT-4, an AI text generative model, in the context of medical text synthesis. By employing a systematic methodology and retrieving information from international guidelines on DKA, we successfully demonstrated the benefits of integrating ChatGPT-4 with the 'Link Reader' plug-in. Clear prompting instructions and a step-wise decomposition of the task strengthened the approach. Despite some limitations, such as occasional memory drift and challenges in accessing web pages, our system maintained a commendable level of accuracy and reliability. The findings of this study hold significant implications for AI-assisted medical research. By providing a framework for automating medical text synthesis and addressing limitations, our work contributes to developing AI text generative models as valuable tools for medical professionals and researchers. Further advancements in this field have the potential to enhance the utility and applicability of AI-generated recommendations in scientific academia. Continued research and refinement of AI models will be crucial in unlocking their full potential and driving advancements in medical research.

## Appendices

Appendix 1

What are the diagnostic criteria for DKA?

Hyperglycemia: All three guidelines agree that a high blood glucose level is a key diagnostic criterion for DKA. The specific threshold varies, with one guideline specifying a blood glucose level of >11 mmol/L [12], another specifying typically ≥14.0 mmol/L but can be lower, especially with the use of SGLT2 inhibitors [11], and the third guideline not providing a specific numerical value but stating that capillary blood glucose should be above 11 mmol/L [10].

Acidosis: All three guidelines agree that metabolic acidosis is a key diagnostic criterion for DKA. The specific thresholds for venous pH and bicarbonate levels vary slightly, with one guideline specifying a venous pH less than 7.3 and/or bicarbonate less than 15 mmol/L [10], another specifying typically an arterial pH ≤7.3, serum bicarbonate ≤15 mmol/L, and an anion gap >12 mmol/L [11], and the third guideline specifying a venous pH <7.3 or bicarbonate <15 mmol/L [12].

Ketones: All three guidelines agree that the presence of ketones, either in the blood or urine, is a key diagnostic criterion for DKA. The specific thresholds for ketone levels vary, with one guideline specifying capillary ketones above 3 mmol/L or urine ketones ++ or more [10], another specifying the presence of serum and/or urine ketones without providing a specific numerical value [11], and the third guideline specifying the presence of blood ketones or urinary ketones (abnormal ketone level is ≥0.6 mmol/L, severe ketosis is >3.0 mmol/L) [12].

Recommendation: Based on the above analysis, the most common diagnostic criteria for DKA can be summarized as follows: Hyperglycemia (typically blood glucose level >11 mmol/L), metabolic acidosis (typically venous pH <7.3 and/or bicarbonate <15 mmol/L), and the presence of ketones in the blood or urine. It is important to note that these criteria may vary slightly depending on specific patient circumstances, such as the use of SGLT2 inhibitors, and should always be interpreted in the context of the patient's overall clinical picture.

What are the risk factors associated with Diabetic Ketoacidosis (DKA)?

The risk factors for Diabetic Ketoacidosis (DKA) across the three guidelines are largely consistent, with minor variations in the specific factors. The common risk factors include:

New diagnosis of diabetes mellitus: This is a common risk factor mentioned among the guidelines [11].

Insulin omission: This is a common risk factor mentioned among the guidelines [11].

Infection: This is a common risk factor mentioned among the guidelines [11].

Use of certain medications: The use of certain medications, including high-dose steroids and sodium glucose co-transporter 2 (SGLT2) inhibitors, is mentioned as a risk factor in one of the guidelines [12].

Stress conditions: Stress conditions such as surgery, trauma, and infections are mentioned as risk factors in one of the guidelines [12].

Recommendation: Based on the above analysis, the most common risk factors for DKA can be summarized as follows: new diagnosis of diabetes mellitus, insulin omission, infection, use of certain medications, and stress conditions such as surgery, trauma, and infections. It is important to note that these risk factors may vary slightly depending on specific patient circumstances, such as the use of SGLT2 inhibitors, and should always be interpreted in the context of the patient's overall clinical picture. It is recommended that healthcare professionals inform all patients commencing SGLT2 inhibitors about the risks of DKA/euglycaemic DKA, including potential symptoms and signs, and provide management advice [12].

What are the signs and symptoms of DKA?

The signs and symptoms of Diabetic Ketoacidosis (DKA) are consistent across the three guidelines, with some variations in the specific symptoms and signs mentioned. The common symptoms include:

Polyuria: Increased frequency of urination is a common symptom mentioned in two of the guidelines [10][11].

Polydipsia: Increased thirst is a common symptom mentioned in two of the guidelines [10][11].

Nausea and vomiting: These symptoms are mentioned in one of the guidelines [10].

Abdominal pain: This symptom is mentioned in one of the guidelines [10].

Shortness of breath: This symptom is mentioned in one of the guidelines [10].

Confusion or difficulty paying attention: This symptom is mentioned in one of the guidelines [10].

Fatigue or extreme tiredness: This symptom is mentioned in one of the guidelines [10].

The common signs include:

Hyperglycaemia: Defined by a blood glucose level (BGL) >11 mmol/L, this sign is mentioned in one of the guidelines [12].

Acidosis: This sign, characterized by a venous pH <7.3 or bicarbonate <15 mmol/L, is mentioned in one of the guidelines [12].

Presence of blood or urinary ketones: This sign is mentioned in one of the guidelines [12].

Recommendation: Based on the above analysis, the most common signs and symptoms of DKA can be summarized as follows: Polyuria, polydipsia, nausea and vomiting, abdominal pain, shortness of breath, confusion or difficulty paying attention, fatigue or extreme tiredness, hyperglycaemia, acidosis, and the presence of blood or urinary ketones. It is important to note that these symptoms and signs may vary slightly depending on specific patient circumstances and should always be interpreted in

What are the investigations for DKA?

The investigations for Diabetic Ketoacidosis (DKA) are comprehensive and involve several biochemical tests. According to the guidelines, the diagnostic criteria for DKA include hyperglycaemia, defined by a Blood Glucose Level (BGL) greater than 11 mmol/L, venous pH less than 7.3 or bicarbonate less than 15 mmol/L, and the presence of blood or urinary ketones [12].

In addition to these, plasma levels of electrolytes and anion gap, plasma glucose, creatinine, osmolality, beta-hydroxybutyric acid (beta-OHB), blood gases, and serum and urine ketones are also assessed [11]. Fluid balance and level of consciousness are monitored closely, and precipitating factors and complications must be diagnosed and treated [11].

Blood ketone testing is preferred, and the equipment should be made available for medical practices and 'at-risk' patient use [12]. The main aim in treating DKA is to progressively normalise the blood pH and clear the body of excessive ketones, achieved by aggressive fluid replacement and insulin therapy [12].

It is important to note that these investigations are part of the management process for DKA and should be conducted under the supervision of a healthcare professional. The specific investigations may vary based on the individual's condition and the clinical judgment of the healthcare provider [10][11][12].

Recommendation: Given the complexity and potential severity of DKA, it is crucial to conduct a comprehensive set of investigations to ensure accurate diagnosis and effective treatment. These investigations should include, but not be limited to, the diagnostic criteria for DKA, assessment of plasma levels of electrolytes, anion gap, plasma glucose, creatinine, osmolality, beta-OHB, blood gases, serum and urine ketones, fluid balance, and level of consciousness. Additionally, precipitating factors and complications should be diagnosed and treated. Blood ketone testing is preferred, and the equipment should be made available for medical practices and 'at-risk' patient use. The main aim in treating DKA is to progressively normalise the blood pH and clear the body of excessive ketones, achieved by aggressive fluid replacement and insulin therapy. These investigations should be conducted under the supervision of a healthcare professional, and the specific investigations may vary based on the individual's condition and the clinical judgment of the healthcare provider.

What is the treatment for DKA?

The treatment for Diabetic Ketoacidosis (DKA) involves several steps, including fluid resuscitation, insulin administration, avoidance of hypokalemia and rapidly falling serum osmolality, and the search for precipitating causes [11]. Fluid resuscitation is initiated with intravenous 0.9% sodium chloride, initially at 500 mL/h for 4 hours, then 250 mL/h for 4 hours [11]. An infusion of short-acting intravenous insulin of 0.10 units/kg/h should be used, and the insulin infusion rate should be maintained until the resolution of ketosis as measured by the normalization of the plasma anion gap [11].

In addition to these, a fixed rate intravenous insulin infusion (FRIII) is commenced at a rate of 0.1 units/kg/hr based on an estimate of weight [10]. If the patient normally takes long-acting insulin analogues (glargine, detemir, degludec), these should be continued at the usual dose and time [10].

Furthermore, blood ketone testing is preferred, and the equipment should be made available for medical practices and 'at-risk' patient use [12]. The main aim in treating DKA is to progressively normalize the blood pH and clear the body of excessive ketones, achieved by aggressive fluid replacement and insulin therapy [12].

Recommendation: Given the complexity and potential severity of DKA, it is crucial to conduct a comprehensive set of treatments to ensure effective management. These treatments should include fluid resuscitation, insulin administration, avoidance of hypokalemia and rapidly falling serum osmolality, and the search for precipitating causes. Blood ketone testing is preferred, and the equipment should be made available for medical practices and 'at-risk' patient use. The main aim in treating DKA is to progressively normalize the blood pH and clear the body of excessive ketones, achieved by aggressive fluid replacement and insulin therapy. These treatments should be conducted under the supervision of a healthcare professional, and the specific treatments may vary based on the individual's condition and the clinical judgment of the healthcare provider.
